# OLFACTION AND DEMENTIA RISK IN THE ATHEROSCLEROSIS RISK IN COMMUNITIES (ARIC) STUDY

**DOI:** 10.1093/geroni/igae098.2134

**Published:** 2024-12-31

**Authors:** Honglei Chen, Srishti Shrestha, Xiaoqian Zhu, Anna Kucharska-Newton, Yaqun Yuan, Vidyulata Kamath, Tom Mosley

**Affiliations:** Michigan State University, Michigan State University, Michigan, United States; University of Mississippi Medical Center, Jackson, Mississippi, United States; University of Mississippi Medical Center, Jackson, Mississippi, United States; University of North Carolina at Chapel Hill, Chapel Hill, North Carolina, United States; Michigan State University East Lansing, East Lansing, Michigan, United States; Johns Hopkins University School of Medicine, Baltimore, Maryland, United States; University of Mississippi Medical Center, Jackson, Mississippi, United States

## Abstract

We examined the association of olfaction with incident dementia and characterized this relationship by key demographic subgroups and APOE-ε4 genotype in the ARIC study. We further examined how change in olfaction was associated with dementia incidence. Olfaction was evaluated using the 12-item Sniffin’ Sticks test in 4470 ARIC participants in 2011-2013 (visit 5) and in 2658 participants in 2016-2017 (visit 6). We defined olfaction as good (score 11-12), moderate (9-10), hyposmia (7-8), and anosmia (0-6). Dementia status was ascertained from 2011-2013 through 2020. Of the 4470 participants at baseline (age:75±5 years, 21% Black race, 60% women), 17% developed dementia over 10 years. In multivariable Cox models, moderate olfaction (hazard ratio (HR):1.5, 95%CI:1.3-1.9), hyposmia (HR:2.2, 95%CI:1.8-2.8), and anosmia (HR:3.5, 95%CI:2.8-4.3) were associated with higher dementia rate in a linear, monotonic manner relative to good olfaction. Findings were similar across subgroups of age, sex, race, and APOE-ε4 genotype. We also used logistic regression to estimate marginal dementia probability. We found that, on the risk difference scale, the absolute risk difference between anosmia and good olfaction was higher in APOE-ε4 carriers (0.29, 95%CI:0.20-0.37) compared to that in non-carriers (0.16, 95%CI:0.11-0.21); jointly, APOE-ε4 carriers with anosmia had the greatest cumulative dementia probability. Further, compared to participants who maintained good olfaction from visit 5 to visit 6, those with stable anosmia (HR:5.6, 95%CI:2.7-11.6) and a decline from good to anosmia (HR:4.5, 95%CI:2.2-9.4)) showed comparable associations with dementia incidence. Our study provides important data about how simple noninvasive olfaction tests may inform future dementia risk.

